# The Abnormal Expression of Tubular SGLT2 and GULT2 in Diabetes Model Mice with Malocclusion-Induced Hyperglycemia

**DOI:** 10.3390/biomedicines13020267

**Published:** 2025-01-22

**Authors:** Koichiro Kajiwara, Sachio Tamaoki, Yoshihiko Sawa

**Affiliations:** 1 Department of Oral Growth & Development, Fukuoka Dental College, 2-15-Tamura, Sawara-ku, Fukuoka 814-0193, Japan; kajiwak@fdcnet.ac.jp (K.K.); tama@fdcnet.ac.jp (S.T.); 2 Department of Oral Function & Anatomy, Okayama University Graduate School of Medicine, Dentistry and Pharmaceutical Sciences, 2-5-1 Shikata-cho, Kita-ku, Okayama 700-0914, Japan

**Keywords:** malocclusion, hyperglycemia, SGLT2, GLUT2

## Abstract

**Background:** A relationship between malocclusion and the promotion of diabetes has been suggested. In hyperglycemia, the expression of sodium–glucose cotransporter 2 (SGLT2) and the facilitative glucose transporter 2 (GLUT2) is upregulated in proximal tubular cells, leading to an increase in renal glucose reabsorption. The present study aimed to investigate whether malocclusion contributes to diabetic exacerbation. **Methods:** Streptozotocin (STZ)-induced diabetic mice with malocclusion due to cutting molars were investigated based on increased blood glucose levels. PCR and immunohistochemical analyses were performed on diabetic mice kidneys to investigate the expression of SGLT2 and GLUT2. **Results:** Animal experiments were performed using 32 mice for 21 days. The time to reach a diabetic condition in STZ-administered mice was shorter with malocclusion than without malocclusion. The increase and mean blood glucose levels in STZ-administered mice were steeper and higher with malocclusion than without malocclusion. Urea albumin, BUN, and CRE levels were higher in diabetic mice with malocclusion than in diabetic mice without. Immunoreaction with anti-SGLT2 and anti-GLUT2 in the renal tissue of STZ-administered mice was stronger with malocclusion than without malocclusion. The amounts of SGLT2 and GLUT2 mRNA in the renal tissue in STZ-administered mice were higher with malocclusion than without malocclusion. The amounts of TNF-a and IL-6 mRNA in the large intestinal tissue in STZ-administered mice were higher with malocclusion than without malocclusion. **Conclusions:** Our results indicate that malocclusion accelerates the tubular expression of SGLT2 and GLUT2 under hyperglycemia. Malocclusion may be a diabetes-exacerbating factor with increased poor glycemic control due to shortened occlusion time resulting from swallowing food without chewing.

## 1. Introduction

A relationship between the deterioration of occlusal support and the promotion of type 2 diabetes has been suggested, but the biological evidence is unclear. Epidemiological studies have shown that the inefficiency of mastication due to reduced occlusal support of teeth is associated with increased poor glycemic control in patients with diabetes [1,2,3,4]. The number of functional occlusal support areas in the Eichner classification is inversely proportional to blood glucose levels and the prevalence of diabetes. It has also been shown that blood glucose levels may decrease after fixed implant-supported restoration in patients with diabetes with reduced occlusal support [1,2,3,4]. Since malocclusion is prone to blood glucose spikes due to shortened occlusal contact time by swallowing without chewing, it is naturally thought that dental treatment that increases occlusal support may reduce the risk of diabetes exacerbation by improving postprandial glucose metabolism through good mastication and extended mealtime. However, since all previous epidemiological studies have been retrospective, it is not certain whether people developed diabetes due to tooth loss by dental caries and periodontitis because they became susceptible to infection due to diabetes. Furthermore, there have been no in vivo studies investigating whether tooth loss is a risk factor for diabetes and blood glucose spikes.

On the other hand, histological signs of diabetes are beginning to be shown for renal functional molecules. We have shown that the administration of *Porphyromonas gingivalis* lipopolysaccharide (LPS), which is one of the pathogens of periodontal disease under the buccal mucosa, induces the promotion of diabetes with the excessive expression of sodium-glucose cotransporter 2 (SGLT2) in the proximal tubules [5]. Glucose is transported across the luminal membrane by SGLT1 and SGLT2 and released through the basolateral membrane by the facilitative glucose transporters 1 and 2 (GLUT1 and GLUT2) [6,7]. GLUT2 is a main protein that aids in regulating glucose homeostasis in the kidney, and approximately 90% of the renal glucose filtered from glomeruli is reabsorbed at the early proximal tubules by SGLT2. The glucose, which is actively transported by SGLT2 from the tubular lumen into the proximal renal tubule epithelial cells against the concentration gradient, localizes at the basolateral cell border and returns to the renal vein [8,9]. It has been reported that SGLT2 is a critical exacerbation factor because the severity of diabetes increases in a production amount-dependent manner of SGLT2 [6,7,8,9]. Therefore, SGLT2 inhibitors are clinically used to suppress diabetic nephropathy [5]; however, the mechanisms of the abnormal SGLT2 expression are not fully elucidated. In hyperglycemia, renal glucose reabsorption increases depending on the expression levels of SGLT2 and GLUT2 in the proximal tubules [6,7,8,9]. It has been shown that the expression levels of SGLT2 and GLUT2 and glucose transport amounts upregulate in proximal tubular cells in both cultured cells and diabetic models and humans [5,6,7,8,9]. The GLUT2 is usually located on the basolateral side of the renal proximal tubular epithelial cells but is translocated to the apical brush border in hyperglycemia. The glucose accumulated in tubular cells is released into the interstitial space along the concentration gradient by an ATP-independent passive transport, which is coordinated by GLUT2. Therefore, it has been thought that blood glucose at high levels induces the expression of SGLT2 and GLUT2 in the renal proximal tubular cells. The present study aimed to investigate whether malocclusion contributes to diabetic exacerbation due to the rise in blood glucose level by the in vivo analysis, focusing on the overexpression of SGLT and GLUT2 in renal tissue.

## 2. Materials and Methods

### 2.1. Animals

The experimental protocol was approved by the Animal Experiment Committee of Fukuoka Dental College (No. 19010) and conducted in the Fukuoka Dental College Animal Center following conditions described elsewhere. Eight-week-old male ICR (Kyudo, Fukuoka, Japan) mice that were genetically identical were used according to the guidelines of the Animal Experiment Committee of Fukuoka Dental College based on the viewpoint of animal welfare [6]. This study used 4 groups (8 mice in each group): non-treated healthy control, malocclusion-administered healthy control, diabetic control, and malocclusion-administered diabetic experimental. Daily, we assessed the humane endpoints according to the ARRIVE guidelines, and mice reaching humane endpoints were euthanized by induction anesthesia with intraperitoneal injections of sodium pentobarbital and cervical dislocation. All mice were used as the data included in the experimental and control data, and there were no exclusions. The protocol for making STZ-administered diabetic mice was described elsewhere [6,10,11,12]. Briefly, a single intraperitoneal injection of STZ (200 mg/kg body weight; Sigma-Aldrich Japan, Tokyo, Japan) in a 0.05 M citric acid buffer at pH 4.5 (20 mg/mL) and cutting enamel of cusps of right molars by a diameter 2.3 mm round carbide bur as malocclusion due to the deterioration of occlusal support were simultaneously performed to mice under inhalation anesthesia. Then, the blood glucose concentrations were checked by a Glutest Sensor (Sanwa Kagaku Kenkyusyo Co., Ltd., Nagoya, Japan) twice a week after the STZ administration and malocclusion treatment. Most studies for the STZ-administered murine diabetes model consider blood glucose levels over 300 mg/dL as a sign of diabetes, and 50% of mice usually become diabetic two weeks after STZ administration with blood glucose levels in the over 300 to 600 mg/dL range. In this study, mice whose blood glucose levels reached over 300 mg/dL were determined to be diabetic, and mice whose blood glucose levels reached over 600 mg/dL were used in the renal immunohistochemical study as the severe diabetic model, according to our previous studies. Urine and blood samples of mice were collected after euthanasia on the day the mice reached humane endpoints, and other mice were collected on the day the last diabetic mice reached humane endpoints and at the end of the experimental period (21 days). The samples were analyzed for urine albumin by albumin ELISA kit (Bethyl Laboratories, Inc., Montgomery, TX, USA), for blood urea nitrogen (BUN) by DetectX (BUN detection kit, Arbor Assays LLC, Ann Arbor, MI, USA), and for blood creatinine (CRE) by LabAssay (Fujifilm Wako Pure Chemical Corporation, Osaka, Japan).

### 2.2. Real-Time PCR

For the mRNA expression amounts of SGLT2 and GLUT2 in the mouse kidney tissue and of TNF-α and interleukin (IL)-6 in the mouse gingival tissue around the teeth with mal-occlusion and large intestine, the real-time PCR was conducted by primer sets where the specificities had been confirmed by the manufacturer (Sigma-Aldrich Japan) described elsewhere (Table 1) [13]. Immediately after excision, 5 mm tissue squares were ground into a paste with a scalpel on glass plates on ice and dissolved in the RLT buffer of an RNeasy kit (Qiagen, Inc., Tokyo, Japan). The total RNA extraction was performed with a QIAshredder column and an RNeasy kit (Qiagen). When many non-specific bands were identified at the gel electrophoresis after the PCR, a DNAfree kit (Ambion, Huntingdon, UK) was used to remove contaminating genomic DNA. The cDNA samples were analyzed by RT-PCR to quantify the mRNA amounts with 50 pM of primer sets. The cDNA (1 μL) was amplified in a 25 μL volume of PowerSYBR Green PCR Master Mix (Applied Biosystems, Foster City, CA, USA) in a Stratagene Mx3000P real-time PCR system (Agilent Technologies, Inc., Santa Clara, CA, USA) and the fluorescence was monitored at each cycle. The β-actin/tested gene cDNA levels in each sample were quantified against β-actin/tested gene standard curves by allowing the Mx3000P 4.10 software to accurately determine each cDNA unit. Finally, the target gene cDNA amounts in each sample were normalized to β-actin cDNA. All data were normalized to controls with no treatment and expressed in arbitrary units.

### 2.3. Immunohistochemistry

Normal kidney tissue confirmed by hematoxylin and eosin staining to have no histological abnormalities was used [13]. The protocol was according to the method described elsewhere [13]. The frozen 10 μm mouse renal tissue sections cut in a cryostat on the sliding glass were fixed in 100% methanol for 5 min at −20 °C. The sections were rinsed with 10 mM PBS and then immersed in the PBS-blocking solution containing goat serum (0.1%) for 30 min at 20 °C. The sections were immunostained with a rabbit polyclonal anti-mouse SGLT2 (#ab85626, Abcam plc., Cambridge, UK) to discriminate proximal tubules, with a hamster monoclonal anti-mouse podoplanin clone 8.8.1 (#127402, BioLegend Inc., San Diego, CA, USA) to distinguish renal glomerular podocytes, and with a rabbit polyclonal anti-mouse GLUT2 (#ab54460, Abcam). The sections were treated with a blocking solution containing primary antibodies (1 µg/mL) for 8 h at 4 °C. After the treatment, the sections were washed three times in PBS for 10 min and immunostained for 1 h at 20 °C with Alexa Fluor 488/568-conjugated goat anti-hamster/rabbit IgGs (0.1 μg/mL, Probes Invitrogen Com., Eugene, OR, USA). The sections were mounted in 50% polyvinylpyrrolidone solution and examined by microscope digital camera systems with CFI Plan Apo Lambda lens series and DS-Ri2/Qi2 (Nikon Corp., Tokyo, Japan). All experiments were repeated three times.

### 2.4. Measurements of the Immunostained Areas of Tissue Sections

The glomeruli and proximal convoluted tubules immunostained by anti-SGLT2 (Abcam) and anti-mouse GLUT2 (R&D Systems, Minneapolis, MN, USA) were counted by using fluorescence microscopy (BZ-810; Keyence Corp., Osaka, Japan). Relative numbers of the SGLT-2-positive organs were expressed as arbitrary units according to the formula of the normalization to ICR healthy control: the number of organs in ICR healthy control with malocclusion, diabetic mice, or diabetic mice with malocclusion/ICR healthy control. All experiments were repeated at least five times.

### 2.5. Statistics

Animal experiments were performed with 32 mice from 4 groups (8 mice in each group) as described above. All experiments for immunohistochemistry and RT-PCR were repeated five times. Data were expressed as the mean + SD and mean values were calculated with standard deviations. The statistical significance of differences (*p* < 0.01) was determined by one-way ANOVA and two-tailed unpaired Student’s *t*-test with STATVIEW 4.51 software (Abacus concepts, Calabasas, CA, USA). The corresponding author is fully aware of the group allocation at the different stages of the experiments. The data analysis and assessments were performed by all co-authors. To prevent any subjective bias of the data evaluators from affecting the observation results, the analysis method was designed so that the evaluators did not know which experimental group’s tissue they were evaluating. In other words, the validity of the data evaluation was confirmed five times using a double-blind method in which the observing researchers themselves did not know the analysis results.

## 3. Results

### 3.1. Changes in Blood Glucose Levels

**Figure 1:**
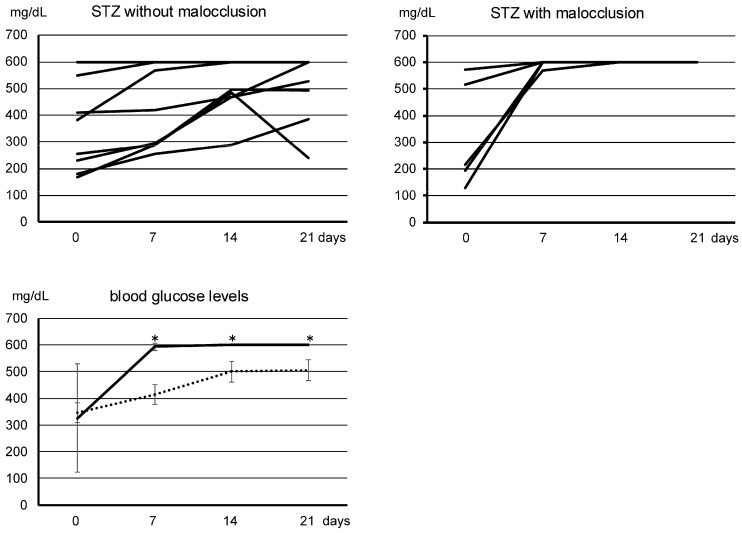
Quantitative analysis of blood sugar levels. The increased ratio of blood glucose levels measured every seven days (d) varied widely in the STZ-administered mice without malocclusion, whereas all STZ-administered mice with malocclusion reached a severe diabetic state over 600 mmHg/dL (top panels). The time to reach a diabetic condition in which the blood glucose level was over 300 mg/dL in STZ-administered mice was shorter with malocclusion than without malocclusion. The increase in the blood glucose level in STZ-administered mice was significantly steeper with malocclusion than without malocclusion. The mean blood glucose levels in STZ-administered mice were statistically significantly higher with malocclusion (solid line) than without malocclusion (dotted line). * Significantly different from the other three in ANOVA (*p* < 0.001). Values are mean ± SE.

**Figure 2:**
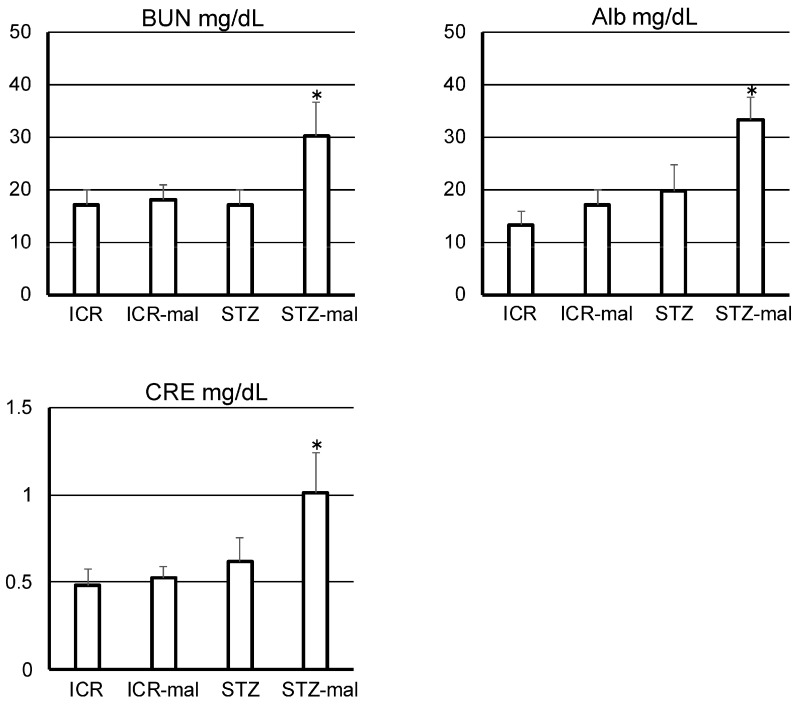
Quantitative analysis of nephrology in diabetic mice with malocclusion. Urine and blood samples of STZ-administered diabetic mice with malocclusion (STZ-mal) were collected after euthanasia on the day the mice reached humane endpoints; samples of healthy mice (ICR), healthy mice with mal occlusion (ICR-mal), and STZ-administered diabetic mice (STZ) were collected on the day the last diabetic mouse with malocclusion reached humane endpoints. The samples were analyzed for urine albumin, blood urea nitrogen (BUN), and blood creatinine (CRE). Albumin, BUN, and CRE levels were significantly higher in the diabetic mice administered malocclusion than in the diabetic mice not administered malocclusion, as well as the other mice. * Significantly different compared to the other three in ANOVA (*p* < 0.001). Values are mean ± SE.

When STZ was simply administered according to conventional methods, the initial increase in blood glucose levels varied widely, but all mice with malocclusion fell into a severe diabetic state with levels of over 600 mmHg/dL (Figure 1). The time to reach a diabetic condition in which the blood glucose level was over 300 mg/dl in STZ-administered mice was shorter with malocclusion than without malocclusion, and the increase in blood glucose level in STZ-administered mice was significantly steeper with malocclusion than without malocclusion (Figure 1). The mean blood glucose levels in STZ-administered mice were statistically significantly higher with malocclusion than without malocclusion (Figure 1). In the quantitative analysis of nephrology in diabetic mice with malocclusion, it was shown that urea albumin, BUN, and CRE levels were significantly higher in diabetic mice administered malocclusion than in diabetic mice not administered malocclusion (Figure 2).

### 3.2. Expression of SGLT2 and GULT2 in the Mouse Renal Tissue

**Figure 3:**
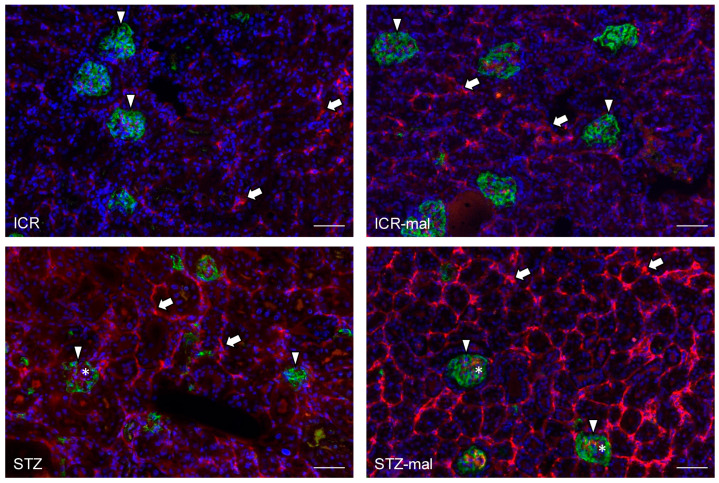
Expression of SGLT2 in the renal tissue of the mice. The tissue sections were immunostained by anti-SGLT2 in red (arrows) and anti-podoplanin to demonstrate glomeruli in green (arrowheads). The sections were counterstained to nuclei by DAPI in blue. The reaction with anti-SGLT2 was observed in healthy mice (ICR) and in healthy mice with malocclusion (ICR-mal), whereas a strong reaction with anti-SGLT2 was observed in STZ-administered diabetic mice (STZ) and diabetic mice with malocclusion (STZ-mal) at a stronger extent than without malocclusion. In diabetic mice, the reaction products with anti-SGLT2 were observed not only in renal proximal tubules but also glomeruli (asterisks). Bar: 100 μm.

**Figure 4:**
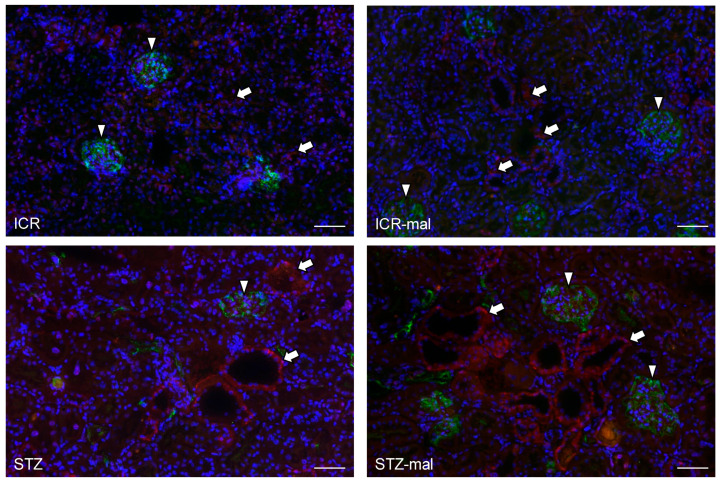
Expression of GLUT2 in the renal tissue of the mice. The tissue sections were immunostained by anti-GLUT2 in red (arrows) and anti-podoplanin to demonstrate glomeruli in green (arrowheads). The sections were counterstained to nuclei by DAPI in blue. The reaction with anti-GLUT2 was rarely observed in control mice without any treatment (ICR) and in mice with malocclusion (ICR-mal), whereas a strong reaction with anti-GLUT2 was observed in the STZ-administered diabetic mice (STZ) and the diabetic mice with malocclusion (STZ-mal) at a stronger extent than without malocclusion. In the STZ-administered diabetic mice, the reaction products with anti-SGLT2 were not observed in glomeruli. Bar: 100 μm.

**Figure 5:**
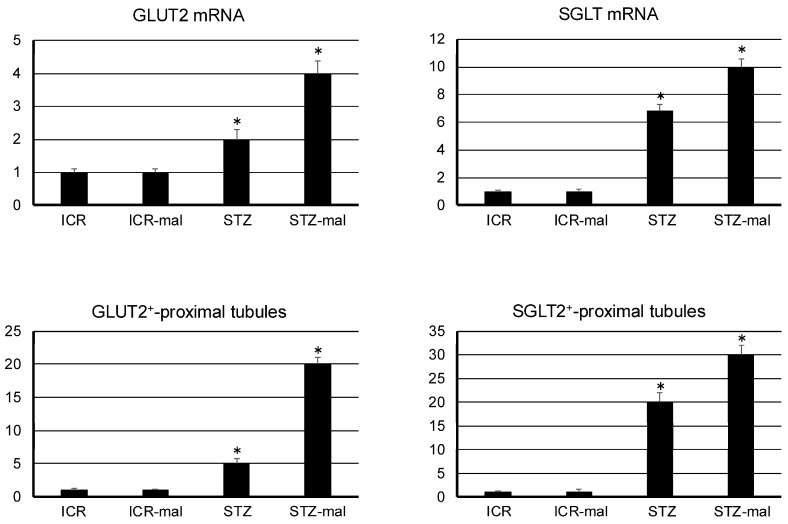
Quantitative analysis for SGLT2 and GLUT2 in the renal tissue in the mice. Top panels: mRNA production. Both SGLT2 and GLUT2 demonstrated higher amounts in the STZ-administered mice with malocclusion (STZ-mal) than in healthy mice (ICR), in healthy mice with malocclusion (ICR-mal), and STZ-administered mice without malocclusion (STZ). The tested gene cDNA amounts were normalized to β-actin cDNA, and all data were expressed relative to controls in arbitrary units. Bottom panels: numbers of renal proximal tubules reacted with anti-SGLT2 and anti-GLUT2. Both had higher levels in the STZ-administered mice with malocclusion than in healthy mice, in mice with malocclusion, and in the STZ-administered mice without malocclusion. All data were expressed relative to controls in arbitrary units. * Significantly different compared to the other three in ANOVA (*p* < 0.001).

The immunoreaction with anti-SGLT2 in the renal tissue was observed at the same level in mice both with no treatment and with malocclusion, whereas the immunoreaction was stronger in STZ-administered mice than in mice without STZ administration, and the immunoreaction in the STZ-administered mice was stronger with malocclusion than without malocclusion (Figure 3). In the STZ-administered mice with malocclusion, the reaction products with anti-SGLT2 were observed in the glomeruli and renal proximal tubules (Figure 3). The immunoreaction with anti-GLUT2 in the renal tissue was rarely observed in mice both with no treatment and with malocclusion, whereas the immunoreaction was observed in STZ-administered mice, and the immunoreaction in STZ-administered mice was stronger with malocclusion than without malocclusion (Figure 4). In the STZ-administered mice with malocclusion, the reaction products with anti-GLUT2 were observed in the renal proximal tubules but not in the glomeruli (Figure 4). In the quantitative analysis for the SGLT2 and GLUT2 expression in the mouse renal tissue, the mRNA production amounts of both SGLT2 and GLUT2 were higher in the STZ-administered mice than in healthy mice, and in healthy mice with malocclusion and in the STZ-administered mice it was higher with malocclusion than without malocclusion (Figure 5). Both numbers of renal proximal tubules that reacted with anti-SGLT2 and anti-GLUT2 were higher in the STZ-administered mice than in healthy mice, and in healthy mice with malocclusion, and in the STZ-administered mice, it was higher with malocclusion than without malocclusion (Figure 5).

### 3.3. Gene Expressions of TNF-α and IL-6 in Gingival Tissue with Malocclusion and Large Intestine

**Figure 6:**
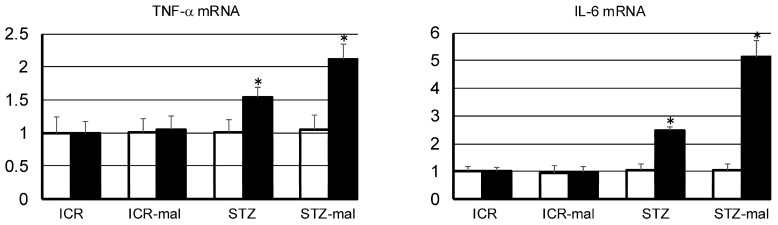
Quantitative analysis for TNF-α and IL-6 mRNA in gingival and large intestinal tissue of mice. For TNF-α and IL-6 mRNA production in the gingival tissue around the teeth with/without malocclusion (open bars), there were no differences in healthy mice (ICR), healthy mice with malocclusion (ICR-mal), STZ-administered mice (STZ), and STZ-administered mice with malocclusion (STZ-mal), whereas in large intestinal tissue (closed bars), STZ-administered mice and STZ-administered mice with malocclusion (STZ-mal) demonstrated more differences than healthy mice (ICR) and healthy mice with malocclusion (ICR-mal). Also, STZ-administered mice with malocclusion (STZ-mal) demonstrated higher amounts than STZ-administered mice. The tested mRNA amounts were normalized to β-actin cDNA, and all data were expressed relative to the controls in arbitrary units. * Significantly different compared to the other three via ANOVA (*p* < 0.001).

For TNF-α and IL-6 mRNA in the gingival tissue around the teeth with/without mal-occlusion (Figure 6), there were no differences in healthy mice, healthy mice with malocclusion, STZ-administered mice, and STZ-administered mice with malocclusion. For TNF-α and IL-6 mRNA production in large intestinal tissue (Figure 6), STZ-administered mice and STZ-administered mice with malocclusion demonstrated higher amounts than healthy mice and healthy mice with malocclusion, and STZ-administered mice with malocclusion demonstrated higher amounts than STZ-administered mice.

## 4. Discussion

### 4.1. Effect of Malocclusion on Blood Glucose Levels

There are many reports suggesting that malocclusion is an important risk factor for the exacerbation of diabetes from the standpoint of blood sugar spikes, an abnormally rapid surge in blood glucose after eating (1–5). In this study, in STZ-administered mice with malocclusion, an increase in blood sugar shortened the time to reach a diabetic condition over 300 mg/dL. This steep increase in blood glucose levels increased the mean blood glucose levels in STZ-administered mice, compared with usual diabetic models of mice (Figure 1). These findings suggest that malocclusion influences the regulation of blood glucose in diabetic conditions. Interestingly, the increased ratio of blood glucose levels varied widely in the STZ-administered mice without malocclusion, whereas all the STZ-administered mice with malocclusion reached a severe diabetic state of over 600 mmHg/dL. This may suggest that malocclusion accelerates the exacerbation of diabetes. It has been reported that uncontrolled diabetes levels are higher in patients with malocclusion than in patients without malocclusion. It is thought that malocclusion is an exacerbation factor of diabetes resulting from blood glucose spikes due to swallowing without enough mastication. However, the experimental diabetes caused by STZ-induced β-cell necrosis limits this inference because the model cannot deny the possibility of off-target effects of STZ on organs other than the pancreas. Therefore, future research will require the deletion of the SGLT2/GLUT2 genes in mice with a genetic background that develops diabetes when fed a high-calorie diet, such as KK/Ta-HF mice [13].

### 4.2. Effect of Malocclusion on the SGLT2 and GULT2 Expression in the Renal Tissue

Diabetic mice given afunctional occlusion showed a deterioration of renal function for urea albumin, BUN, and CRE, suggesting that malocclusion affects renal health in diabetic conditions (Figure 2). There were no differences in the weak immunoreaction with anti-SGLT2 between untreated mice and mice with malocclusion (Figure 3), suggesting that malocclusion does not influence the expression of SGLT-2 under healthy conditions. Immunoreaction with anti-SGLT2 was significantly stronger in STZ-administered mice than in healthy mice (Figure 3), suggesting that hyperglycemia increases SGLT-2 expression, as previous studies have indicated (6–9). Immunoreaction with anti-SGLT2 in STZ-administered mice was significantly stronger with malocclusion than without malocclusion, suggesting that malocclusion increases the expression of SGLT-2 under hyperglycemia conditions. There were no differences in rare immunoreaction with anti-GULT2 between untreated mice and mice with malocclusion (Figure 4), suggesting that malocclusion does not influence the expression of GULT2 under healthy conditions. Immunoreaction with anti-GULT2 was significantly stronger in STZ-administered mice than in healthy mice, suggesting that hyperglycemia increases GULT2 expression, as previous studies have indicated (1–6). Immunoreaction with anti-SGLT2 in STZ-administered mice was significantly stronger with malocclusion than without malocclusion, suggesting that malocclusion increases GULT2 expression under hyperglycemia circumstances.

In our quantitative analysis, there were no differences in the renal mRNA SGLT2 and GLUT2 expressions between untreated mice and mice with malocclusion (Figure 5), suggesting that malocclusion does not influence the gene expressions of SGLT2 and GLUT2 under healthy conditions. Renal mRNA SGLT2 and GLUT2 levels were significantly stronger in STZ-administered mice than in healthy mice with/without malocclusion (Figure 5). Both immunohistochemically detectable numbers of renal proximal tubules with SGLT2 and GLUT2 expression were significantly higher in STZ-administered mice than in healthy mice with/without malocclusion (Figure 5). These findings suggest that hyperglycemia increases the expression of SGLT2 and GLUT2, as previous studies have indicated (1–6). Renal mRNA SGLT2 and GLUT2 in STZ-administered mice were significantly stronger with malocclusion than without malocclusion (Figure 5). Both quantities of renal proximal tubules with SGLT2 and GLUT2 expressions in STZ-administered mice were significantly greater with malocclusion than without malocclusion (Figure 5). These findings suggest that malocclusion induces the expression of SGLT2 and GLUT2 under hyperglycemia conditions.

There have been many reports that diabetes contributes to the aggravation of oral diseases and that, conversely, oral diseases contribute to the aggravation of diabetes [14,15]. Furthermore, there are epidemiological reports showing that oral treatment contributes to blood sugar control and that this is probably because inflammatory factors produced in oral tissues induce insulin resistance, but this mechanism has not been fully elucidated. To date, there have been no reports of the effect of occlusal dysfunction on the exacerbation of diabetes using animal experiments. Since diabetic intestinal dysfunction is well known [16], it is thought that occlusal dysfunction caused by dental caries and periodontal disease may worsen diabetes not only by chronic inflammation in the oral cavity but also via chronic fatigue of the digestive tract due to poor chewing. For TNF-α and IL-6 mRNA in the gingival tissue around the teeth with/without malocclusion (Figure 6), there were no differences in healthy mice, mice with malocclusion, STZ-administered mice, or STZ-administered mice with malocclusion, suggesting that the malocclusion treatment in this study has not resulted in gingival inflammation. Remarkably, in the large intestine, malocclusion increased the expression of TNF-α and IL-6 in STZ-administered mice (Figure 6). Since the gene expressions were higher in STZ-administered mice than in healthy mice, the possibility of pharmacological and diabetic effects of STZ cannot be excluded. However, the expression of TNF-α and IL-6 genes increased in STZ-administered mice without the enhancement of the gene expression in gingival tissue; it might be suggested that malocclusion increased the expression of TNF-α and IL-6 genes in the large intestine.

SGLT-2 and GLUT2 are responsible for glucose reabsorption in the renal proximal tubules to maintain glucose homeostasis, and the transcription is upregulated in diabetes due to the glucose-stimulated upregulation in response to the increase in transcription activator hepatic nuclear factor 1-alpha (Hnf1α) which directly binds 5′-flanking region of Sglt2 and Glut2 gene upstream. It has been established that renal Sglt2 and Glut2 gene expression crosstalk in regulating systemic glucose homeostasis depends on renal Hnf1α [9,17,18,19]. It has also been reported that IL-6 upregulates Hnf1α and that SGLT2 increases by IL-6 and TNF-α in cultured kidney epithelial cells [20,21]. Therefore, it is thought that inflammatory cytokines are produced due to the chronic inflammation by intestinal bacteria in the intestines, which are fatigued due to indigestion caused by malocclusion, and enter the kidneys via the circulation and promote the activation of the common transcription factor Hnf1α, which is responsible for SGLT2/GLUT2 expression in the renal tubules. Further investigation for the activation of Hnf1α in the kidney with oral dysfunction is required.

Altogether, this study may suggest that malocclusion under diabetic circumstances could abnormally upregulate SGLT2 and GLUT2 expression in the proximal tubular epithelial cells by sensing blood glucose spikes due to shortened occlusion time by swallowing without chewing [22]. Since susceptibility to blood glucose increases under diabetic conditions and the expressions of SGLT2 and GLUT2 are increased in patients with diabetes with persistent hyperglycemia, malocclusion may be one of the most serious exacerbating factors for not only diabetes but also diabetic nephropathy in patients with reduced occlusal support conditions associated with increased poor glycemic control in patients with diabetes.

## 5. Conclusions

Our results suggest that malocclusion, due to reduced occlusal support, accelerates the expression of SGLT2 and GLUT2 in the proximal tubules under hyperglycemia conditions. Addressing malocclusion may prevent blood glucose spikes and the exacerbation of diabetes by suppressing renal SGLT2 and GLUT2.

## Figures and Tables

**Table 1:** Primer sets for mouse RT-PCR.

Proteins	bp	Upper (5′–3′)	Lower (5′–3′)	NM
GLUT2	217	GATAAATTCGCCTGGATGAGTTACG	GCCCAAGGAAGTCCGCAATG	031197
SGLT2	209	CCCATCCCTCAGAAGCATCTCC	CTCATCCCACAGAACCAAAGCA	133254
β-actin	411	GTTCTACAAATGTGGCTGAGGA	ATTGGTCTCAAGTCAGTGTACAG	7393
IL-6	263	ATGTTCTCTGGGAAATCGTGGAAAT	TCTCTGAAGGACTCTGGCTTTGT	031168
TNF-α	355	GCGAGGACAGCAAGGGACT	GAGGCCATTTGGGAACTTCTCAT	013693

## Data Availability

The datasets generated and/or analyzed during the current study are not publicly available due to the need to keep them confidential but are available from the corresponding author upon request.
